# Formal reply to “Alternative lengthening of telomeres is not synonymous with mutations in ATRX/DAXX”

**DOI:** 10.1038/s41467-021-21796-y

**Published:** 2021-03-10

**Authors:** Lars Feuerbach

**Affiliations:** Division of Applied Bioinformatics, German Cancer Research Center (DKFZ), Heidelberg, Germany

**Keywords:** Cancer genetics, DNA damage and repair, Diagnostic markers

**Replying to** R. R. Reddel and A. de Nonneville. *Nature Communications* 10.1038/s41467-021-21794-0 (2021)

The study of Nonneville and Reddel partially overcomes a limitation of our original work^1^. Namely, it integrates the in silico analysis of the genomic footprints of telomere maintenance mechanisms (TMMs) of the Pan-Cancer Analysis of Whole Genomes (PCAWG) dataset^1^ with molecular assays of ALT status based on C-circle assays.

As PCAWG was in its essence a computational reanalysis of existing data from the ICGC and TCGA projects, generation of such gold standard molecular data was outside of the project’s scope. In consequence, we compared truncating mutations of the *ATRX* and *DAXX* genes against activating mutations of *TERT* to pick out strong representatives of the two classes of TMMs that were identifiable by sequencing data alone and removed all other samples from the training set of the machine learning procedure.

The data that Nonneville and Reddel now used was published after we submitted our study early in 2017 to the internal revision process of the PCAWG consortium, and thus could not be included in our work. Therefore, the current manuscript is a great opportunity to learn more about ALT-positive cancers that lack genetic mutations in *ATRX* or *DAXX*.

The Nonneville and Reddel study use C-circle assays for 167 pancreatic neuroendocrine tumors (PaNET) and melanomas, roughly 6% of the full PCAWG dataset, to establish a molecular gold standard for the ALT status, assuming a one-to-one correspondence of C-circle positive signals and ALT-positive status. They then integrate this data with the genomic footprints that were derived by us using the TelomereHunter software^2^.

Strikingly, they find that five of the features identified in the original study are sufficient to train classifiers that perfectly separates C-circle positive from C-circle negative cases if trained cohort wise (Table 1). Besides telomere content, all these features are singleton telomere variant repeat counts, including the, before the Sieverling et al. study uncharacterized, depletion of TTTGGG singletons and the enrichment of TTCGGG singletons.

**Table 1:** Comparison of features for ALT, respectively, ATRX/DAXX^trunc^ prediction.

Feature name	Matters Arising	Sieverling et al.^1^
TTTGGG singleton divergence to expected count	Included	13.59
TTCGGG singleton divergence to expected count	Included	11.92
Breakpoint count	Not included	11.01
Telomere insertion count	Not included	10.03
Telomere content tumor/control log2 ratio	Included	5.34
TGAGGG singleton divergence to expected count	Not included	5.02
TCAGGG singleton divergence to expected count	Not included	3.25
TTGGGG singleton divergence to expected count	Included	2.83
GTAGGG singleton divergence to expected count	Included	Not included

For each feature, the Matters Arising study provides the qualitative information if the feature has passed the Akaike information criterion. The Sieverling et al. study provides a quantitative measure of feature importance derived from the Random Forest classifier^1^.

Not surprisingly, they report that combining melanoma and PaNET cases for classifier training reduces the accuracy to 93–94%. This is likely due to the combination of increasing biological and technical heterogeneity, as not only the baseline frequencies of genomic alterations differ substantially between melanoma and PaNET, but different tumor cellularity and batch effects during the generation of the sequencing data also impact the underlying data. Interestingly, using Lee et al.^3^ C-circle assay data as target variable in a novel ROC-curve analysis detects also for the ALT probability score proposed in Sieverling et al.^1^ a robust performance (Fig. 1). Choosing a comparable sensitivity-specificity trade-off for both models, the ALT probability model reaches at a specificity level of 93% a sensitivity of 87% which is close to the 93.98% sensitivity reported for Nonneville et al. classifier. This corresponds to a score threshold of 0.5, in contrast to the arbitrarily chosen threshold of 0.75 in Nonneville et al. Separating the melanoma and PaNET cases supports the necessity of cancer-type specific classifiers, as the C-circle status of the former is predict far less accurately by the ALT probability score than the latter (Fig. 1).

**Fig. 1:**
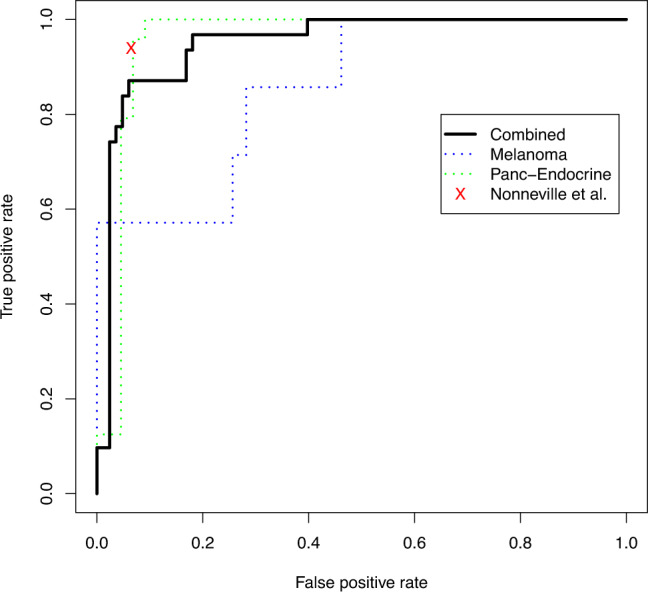
Receiver operating characteristic analysis. The curve shows the True positive rate (sensitivity) and the Fales positive rate (1 – specificity) for all possible thresholds on the ALT probability score (Sieverling et al.^1^). The predicted class label is the C-circle status reported from Lee et al., while the red cross depicts the performance of the classifier proposed in the Matters Arising article for which classification results are reported for only one threshold.

In their study, Nonneville and Reddel suggest that TVR counts correlate with ATRX/DAXX^trunc^ regardless of CCA result. Remarkably, their dataset only contains three ATRX/DAXX^trunc^ samples that are C-circle-negative. As depicted in Fig. 1, indeed two of these strongly derivate in all TVR counts, but drawing such a general conclusion from two out of three observations may be premature. Therefore, it is necessary to either conduct additional ALT assays, such as APB-staining or telomere FISH, to generate an undebatable gold standard for these three samples, and to collect more data from the “C-circle-”/“ATRX/DAXX^trunc^ + ” subgroup to further substantiate this hypothesis. The presented data on the TTCGGG singleton count raises another interesting question. Figure 1 of Nonneville and Reddel shows that not only all “C-circle + ”/“ATRX/DAXX^trunc^ + ” but also about 40% of the “C-circle + ”/“ATRX/DAXX^trunc^ – ” cases show a clear enrichment of TTCGGG singleton counts. If indeed the TVR patterns in general and the TTCGGG pattern, in particular, are linked to the disruption of the ATRX/DAXX complex rather than the ALT status, this would imply for these cancer samples the presence of alterations beyond the genetic mutations that constitute the ATRX/DAXX^trunc^ subgroup, for instance, a downregulation of the complex on the protein level.

Another highly relevant observation is the exclusion of breakpoint and telomere insertion count as features by the Akaike information criterion, which were among the most predictive features in the Sieverling et al. study (Table 1). Does this imply that these features are a mere side effect of ATRX/DAXX disruption and that a more genomically stable ALT subtype with intact ATRX/DAXX complex but increased C-circle levels exist?

The most relevant function of the ATRX/DAXX complex for the ALT phenotype is the maintenance of a condensed chromatin state at the telomeres and elsewhere in the genome. Loss of this function probably increases the likelihood of genomic breaks in the chaperoned loci, and enables the induction of break-induced replication (BIR) that is implied in the recombination of telomeric sequences in ALT^4^.

Future studies integrating multiple omics layers will clarify in how far this function is simply disrupted by additional mechanisms in ALT positive tumors without ATRX/DAXX^trunc^ mutations, and if alternatives with less deleterious impact on genomic stability exist. In any case, the new data emphasizes that ALT positivity does not fully overlap with the ATRX/DAXX^trunc^ annotation. In consequence, the derived score in our original study should be renamed from the ALT probability score into a genomic ATRX/DAXX^trunc^ score to reflect this discovery.

## Methods

The ROC-curve analysis was conducted with the ROCR package^5^. The ATRX/DAXX^trunc^ score, respectively, ALT probability score from Sieverling et al.^1^ was used to evaluate the performance for the individual cohorts and for both cohorts together. As the model in the Matters Arising study does only report a binary classification but no continuous score, the reported performance values of the combined model were marked by a red cross in the diagram. The underlying data is provided as source data in XLSX format.

### Reporting summary

Further information on research design is available in the Nature Research Reporting Summary linked to this article.

## Supplementary information

Reporting Summary

## Data Availability

All used data is available as a supplement to Lee et al.^3^ (Supplementary Table 2), Sieverling et al.^1^ (Supplementary Table 1), and the Matters arising article. For the purpose of patient id mapping between the different datasets the icgc data portal was used: https://dcc.icgc.org/pcawg. Source data are provided with this paper.

## Source data

Source Data
